# Targeting Protein Kinases to Enhance the Response to anti-PD-1/PD-L1 Immunotherapy

**DOI:** 10.3390/ijms20092296

**Published:** 2019-05-09

**Authors:** Marilina García-Aranda, Maximino Redondo

**Affiliations:** 1 Research Unit, Hospital Costa del Sol. Autovía A7, km 187. Marbella, 29603 Málaga, Spain; marilina@hcs.es; 2 Red de Investigación en Servicios de Salud en Enfermedades Crónicas (REDISSEC), 28029 Madrid, Spain; 3 Instituto de Investigación Biomédica de Málaga (IBIMA), 29010 Málaga, Spain; 4 Departamento de Especialidades Quirúrgicas, Bioquímica e Inmunología, Universidad de Málaga, Campus Universitario de Teatinos, 29010 Málaga, Spain

**Keywords:** cancer, kinase, immunotherapy, resistance, checkpoint blockade, tumor escape, inhibitor, MHC

## Abstract

The interaction between programmed cell death protein (PD-1) and its ligand (PD-L1) is one of the main pathways used by some tumors to escape the immune response. In recent years, immunotherapies based on the use of antibodies against PD-1/PD-L1 have been postulated as a great promise for cancer treatment, increasing total survival compared to standard therapy in different tumors. Despite the hopefulness of these results, a significant percentage of patients do not respond to such therapy or will end up evolving toward a progressive disease. Besides their role in PD-L1 expression, altered protein kinases in tumor cells can limit the effectiveness of PD-1/PD-L1 blocking therapies at different levels. In this review, we describe the role of kinases that appear most frequently altered in tumor cells and that can be an impediment for the success of immunotherapies as well as the potential utility of protein kinase inhibitors to enhance the response to such treatments.

## 1. Introduction

### 1.1. Background

Given their ability to destroy aberrant cells, such as pathogen-infected cells or cancer cells, T-cells play a key role in cell-mediated immunity. In order to limit tissue damage and immunologic self-tolerance [1] and according to the two-signal theory of lymphocyte activation [2], this process, which occurs after the recognition of peptide antigen associated to the major histocompatibility complex (MHC/HLA human leukocyte antigen) and presented on targeted cells, is controlled by a series of co-stimulatory and co-inhibitory receptors and their ligands (also known as immune check-points).

Among all the inhibitory immune mediators, the pathway consisting of the programmed cell death protein 1 (PD-1) and its ligands PD-L1 (B7-H1, CD274) and PD-L2 (B7-DC, CD273) [3] has become a relevant tool for the great development that current cancer immunotherapy has achieved in recent years. So much so that PD-1/PD-L1 axis has broadly demonstrated its value as a therapeutic target and its blockade, as a valuable tool to significantly improve patient outcome in a large number of malignancies including melanoma, non-small cell lung cancer (NSCLC), renal cell carcinoma (RCC), Hodgkin’s lymphoma, bladder cancer, head and neck squamous cell carcinoma (HNSCC), Merkel-cell carcinoma, microsatellite instable-high (MSI-H) or mismatch repair-deficient (dMMR) solid tumors [1].

PD-1 is one of the co-inhibitory receptors expressed on the surface of antigen-stimulated T-cells [1]. After binding to PD-1, PD-L1 and PD-L2 exert a repressive activity of the main pathways related to T-cells survival, which dramatically affects T-cell activation and cytokine production, and hence, T-cell capacity to repress tumor cells. Although this interaction is essential to maintain homeostasis of the immune response to prevent autoimmunity during infection or inflammation of normal tissues, in tumor microenvironments it provokes an immune escape for tumor cells through cytotoxic T-cell inactivation. For this reason, the PD-1/PD-L1 pathway blockade has recently become a promising strategy to overcome counteraction and preserve the antitumor capacity of T-cells [4]. 

However, despite the success of this revolutionary discovery and the potent and durable anti-tumor effects in different metastatic tumors, when compared to chemotherapy and molecular targeted therapies, response rates to therapies based on the use of immune checkpoint inhibitors as monotherapy rarely exceeds 40%, ranging between 20%–38% among different tumor types [5,6] and with a significant percentage of partial responders [4] that ranges from 1.3% to 8.3% who end up relapsing [7]. 

On the other hand, resistance to anti-PD-1/PD-L1 therapy is present in up to 70% of patients [8], with up to 60% of patients presenting a primary resistance [8,9], which can be responsible for disease progression or relapse [4] and stresses the need to continue investigating for response biomarkers crucial for patient selection. 

Recent studies in this field have proposed different biomarkers as tools to predict the efficacy of PD-1/PD-L1 inhibitors [10] (Table 1). Among these, PD-L1 status remains as a core predictor for PD-1/PD-L1-blocking patient selection [10]. However, provided that detection of PD-L1 expression alone is not sufficient to predict patient response for most tumor types [11], there is still a need to investigate and develop new immunotherapy biomarker panels along with new treatment strategies to improve the efficacy of such therapies.

### 1.2. Regulation of PD-1 Expression and Activity

Given the decisive role of PD-1 in regulating T-cells exhaustion and therefore, their ability to proliferate, combat cancer cells and synthesize cytokines [12], the study of different factors that can affect the expression of this receptor has aroused a great interest in recent years. 

In humans, PD-1 also known as cluster of differentiation (CD279), human systemic lupus erythematosus (hSLE1) and systemic lupus erythematosus 2 (SLEB2) [1] is encoded by the *PDCD1* gene located at chromosome 2:241,849,881–241,858,908 reverse strand [13]. At least three different transcripts or splice variants (*PDCD1-204*, *PDCD1-206*, *PDCD1-205*) and 80 orthologues [13] of this gene have been described. 

Besides the association between *PDCD1* mutations with disease progression in multiple human autoimmune disorders [13], genetic variants of this gene affect both overall survival and recurrence-free survival of patients with colorectal cancer and hence, would affect the genetic predisposition to an anti-immune reaction in cancer patients [14].

*PDCD1-204* and *PDCD1-205* transcripts are predicted to encode for single-pass type I membrane protein isoforms containing an extracellular domain, a helical transmembrane domain and a cytoplasmic domain [15] with an immunoreceptor tyrosine-based inhibition motif (ITIM) and an immunoreceptor tyrosine-based switch motif (ITSM) [16]. Accordingly, these variants contain several immunoglobulin-like and immunoglobulin V-set domains [17].

In vitro studies on lymphocytic cell lines and in ex vivo stimulated CD8 T-cells have allowed for the characterization of the *PDCD1* gene [12,18] and have demonstrated that PD-1 is temporarily induced on activated CD8 T-cells and constitutively expressed in cells exhibiting the exhausted phenotype [12]. In particular, PD-1 expression can be induced on active T-cells, natural killer T-cells or myeloid cells such as dendritic cells and activated monocytes following T-cell receptor (TCR) activation and stimulation by cytokines as interleukin [19]. 

**Figure 1:**
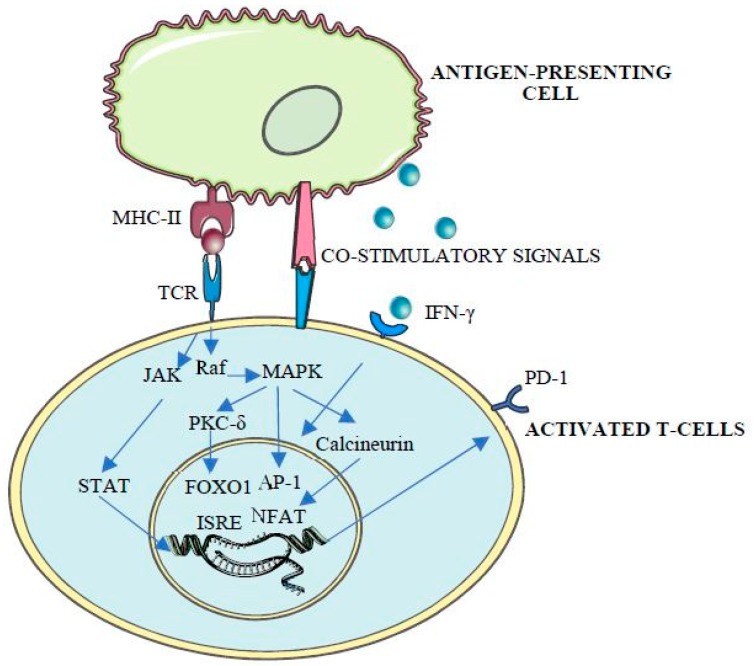
T-cell receptor (TCR) binding to antigen-loaded MHC-II triggers T-cell initial activation. TCR mediates protein kinase C (PKC) and subsequent Raf stimulation, leading to the activation of mitogen-activated protein kinase (MAPK) cascade and AP-1 phosphorylation, NFAT dephosphorylation by calcineurin, FOXO1 phosphorylation and activation by protein kinase C-delta (PKC-δ) and JAK/STAT pathway activation, ultimately leading to PD-1 expression and T-cell activation [20].

Thus, as a mediator of central and peripheral immune tolerance and immune exhaustion [20], *PDCD1* expression is tightly regulated by the combinatorial action of cis-acting elements, including promoters, enhancers, locus control regions and boundary elements [12]. Apart from the first exon (CR-A), sequencing studies show the presence of two highly conserved regions (CR-B and CR-C), located 5’ to the transcriptional start site (TSS) and with strong DNase I hypersensitivity, which suggest a regulatory function of these elements [12]. Consequently, these regions contain both *cis-* and *trans*-acting elements, involved in the transcriptional regulation of PD-1 and associated with CD8T cell activation [12], with binding sites for different transcription factors including activator jun/activator protein (AP)-1, interferon-stimulated response element (ISRE), Nuclear factor of activated T-cells (NFAT)c1, fork-head box protein O1 (FoxO1), nuclear factor (NF)-ĸB, Janus kinase JAK/STAT (signal transducers and activators of transcription) and inhibitory blimp-1 and T-bet [20], whose activity is tightly regulated by protein kinases and phosphatases [20,21,22,23,24,25] (Figure 1).

**Figure 2:**
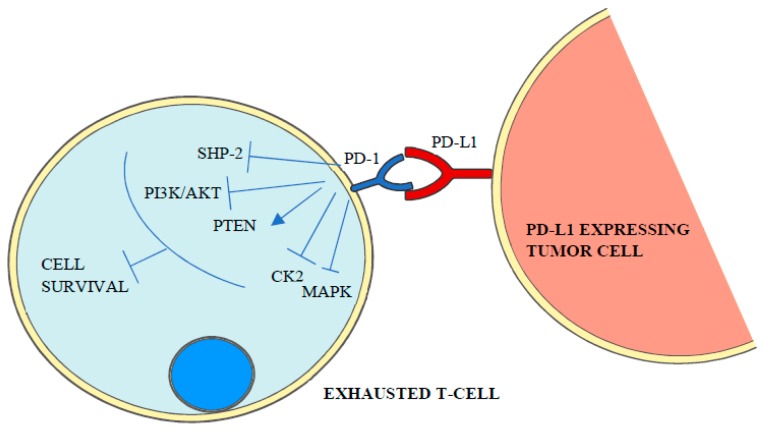
PD-1 binding to its ligand triggers the activation of intracellular signaling cascades that mediate the dephosphorylation and inactivation of T-cell receptor proximal signal components such as SHP-2, PI3K/AKT, MAPK and CK2 leading to lymphocyte exhaustion.

The mechanism of how PD-1 ligands inhibit T-cell receptor signaling, which is under intense study, starts with the phosphorylation of the cytoplasmatic immunoreceptor tyrosine-based inhibitory motif and the immunoreceptor tyrosine-based switch motif by Src kinases and a conformational change that leads to the recruitment of SHP (SRC homology-domain-containing protein tyrosine phosphatase) 1 and 2 [1,3] and the dephosphorylation of T-cell receptor proximal signal components [16] including PI3K/AKT (phosphoinositide 3-kinase/protein kinase B), PTEN (phosphatase and tensin homolog), CK2 (casein kinase 2) and RAS/MEK/ERK (mitogen-activated protein kinase MAPK/extracellular-signal-regulated-kinase) [4]. As a result, T-cell proliferation, survival, cytokine production and other effector functions are inhibited [1] (Figure 2).

### 1.3. Regulation of PD-L1 and PD-L2 Expression

PD-L1, also known as B7-H, B7H1, PDL1, PDCD1L1 or PDCD1LG1, is an immune inhibitory receptor ligand expressed by hematopoietic and non-hematopoietic cells, such as T-cells and B-cells and many types of tumor cells, that in humans is encoded by the *CD274* gene [26]. *CD274* is located at chromosome 9:5,450,503–5,470,566 forward strand, has five transcripts (*CD274-202*, *CD274-201, CD274-205*, *CD274-204*, *CD274-203*), 286 orthologues and 13 paralogues [26]. *CD274-202* and *CD274-201* transcripts encode for single-pass type I transmembrane proteins with immunoglobulin V-like and C-like domains [26]. PD-L1 splice variants lacking transmembrane or intracellular domains and leading to secretion of soluble PD-L1 are under intense study [10], given their role in resistance to PD-L1 blockade therapy [27] and poor prognosis [10].

The other PD-1 ligand, PD-L2, also known as B7DC, Btdc, PDL2, CD273, PD-L2, PDCD1L2, bA574F11.2 [28], is encoded by the *PDCD1LG2* gene located at chromosome 9:5,510,570-5,571,254 forward strand [29], has one splice variant and 120 orthologues [29].

**Figure 3:**
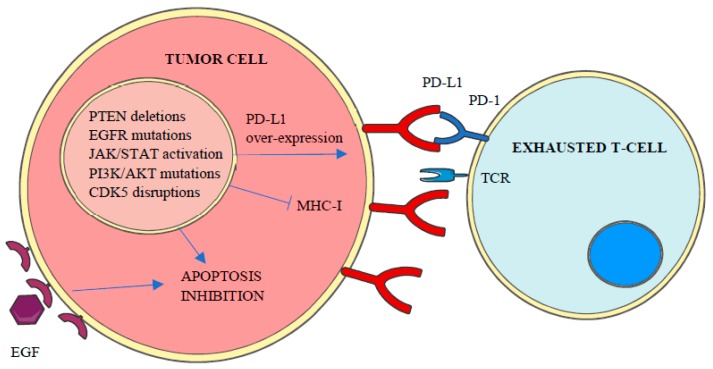
Aberrant expression of different kinases inhibits apoptosis and MHC-I expression and promotes PD-L1 overexpression, which leads to tumor cell enhanced survival and T-Cell inactivation or loss of recognition.

PD-L1 expression in tumor cells can be constitutive or inducible [30] and may vary over time in response to different stimuli such as interferon (IFN)-γ, epidermal growth factor (EGF) or cytokines [10]. In accordance to the repressive activity of PD-L1 and PD-L2 over T-cells, genetic amplifications of *CD274* and *PDCD1LG2* genes have been associated with high local immune cytolytic activity [4] and the enhanced expression of both ligands, with more than 30 different malignancies including lung, melanoma, breast or colon [26,29]. Apart from genetic amplifications and the increase of stabilized PD-L1 transcripts by truncation of *CD274-* 3’ *UTR* [4], PD-L1 over-expression in cancer cells has been related to the aberrant expression of different protein kinases, including constitutive activation of Janus kinase/signal transducers and activators of transcription (JAK/STAT) signaling, PTEN deletions, PI3K and/or AKT mutations, EGF receptor mutations, *MYC* overexpression and cyclin-dependent kinase 5 (CDK5) disruptions [4] (Figure 3).

Apart from the central role of protein kinases on the expression of both PD-1 and its ligands, the aberrant regulation of protein kinase pathways is also a major cause of apoptosis resistance against immune response [31] (Figure 3). Accordingly, different studies have been conducted in recent years exploring the utility of protein kinase inhibitors to enhance the clinical response to anti-PD-1/PD-L1 therapies [32,33,34,35,36,37]. 

### 1.4. Protein Kinases

The aim of both immunotherapy and chemotherapy is the elimination of tumor cells by inducing apoptosis [38]. Even though apoptosis induced by immune responses is regulated by the BCL-2 (B-cell lymphoma-2) family of proteins [39], the expression of these enzymes depends to a large extent on protein kinases activity [31]. Human protein kinases make up a large superfamily, known as the human kinome, of over 500 enzymes that catalyze the reversible transfer of phosphate, diphosphate, nucleotidyl residues and other groups to a receptor molecule [40]. 

In order to better understand the complexity of human kinome, different classification systems have been developed in recent years which are indistinctly used in the current scientific literature. Thus, protein kinases have classically been classified attending to their location in the cell, that is, *Transmembrane Receptor Kinases* consisting on a ligand-binding extracellular domain and a catalytic intracellular kinase domain and *Non-Receptor Kinases* lacking transmembrane domains and located in the cytosol, nucleus or associated to the inner surface of the plasma membrane. On the other hand, the Enzyme List created by the Nomenclature Committee of the International Union of Biochemistry and Molecular Biology includes human protein kinases into the Class 2.7-Transferring phosphorus-containing groups [40] and classifies them into 13 subcategories, being 2.7.10 Tyrosine Kinases and 2.7.11. Serine/Threonine Kinases groups the two major representatives regarding their role as key elements in the regulation on most cellular activities [41]. In 2002 of *The Protein Kinase Complement of the Human Genome* also proposed the classification of human kinases into these 10 main groups regarding catalytic domain sequence comparisons: AGC (A, G and C protein kinases), CAMK (Ca2+/CAM-dependent protein kinases), CK1 (casein kinase 1), CMGC (CDK, cyclin-dependent kinases; MAPK, mitogen-activated protein kinases; GSK3, glycose synthase kinase-3; CLK, cdc2-like kinases), RGC (receptor guanylate cyclase), STE (homologues of yeast sterile 7, 11, 20 kinases), TKs (tyrosine kinases), TKL (tyrosine kinase-like protein kinases), *Other Kinases* group and the *Atypical* group [42]. 

**Figure 4:**
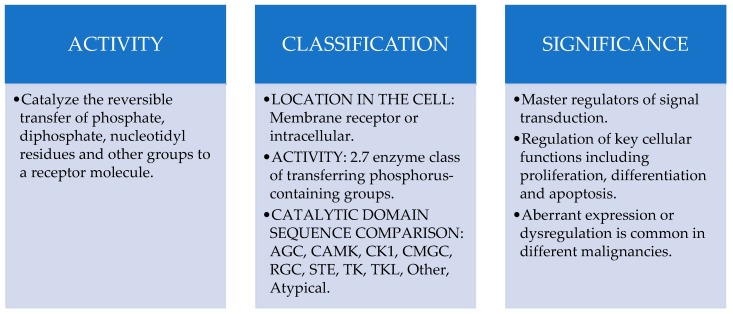
Protein kinases overview.

Along with their agonist phosphatases, these enzymes catalyze one of the most prevalent post-translational modifications involved in the regulation of signal transduction and key cellular functions including proliferation, differentiation and apoptosis [41]. Accordingly, aberrant expression or dysregulation of protein kinases has been reported to be involved in different hallmarks of cancer including proliferation, survival, motility, metabolism, angiogenesis, resistance to standard treatments and immunotherapies [43] and evasion of antitumor immune responses [44], having been demonstrated in the pathophysiology of different malignancies including breast [43], colon, kidney or pancreas [41] (Figure 4). 

The presence of altered kinases represents such a survival advantage over other cells that, under the selective pressure caused by the immune system or chemotherapeutic agents, tumor cell survival becomes dependent on these dysregulated pathways, which ultimately, turn them into the perfect targets for kinase inhibitors. So much so that in recent years more than 25 oncology drugs targeting kinases have been approved and released of [44].

## 2. Role of Protein Kinases in Resistance to Immunotherapy

### 2.1. Primary, Adaptive and Acquired Resistance to Cancer Immunotherapy

Although cancer immunotherapy can induce long-lasting responses in patients with metastatic cancers, in some cases the immune response may be ineffective against a heterogeneous and evolving tumor microenvironment [45]. 

Resistance to cancer immunotherapy has been classified into primary resistance, defined as a lack of response to immunotherapy due to different mechanisms including adaptive immune resistance; adaptive immune response, a mechanism where cancer cells are recognized by the immune system but end up adapting to the immune attack which could lead to primary resistance, mixed responses or acquired resistance and finally, acquired or secondary resistance, which usually occurs after prolonged treatment and in which cancer cells that initially responded to immunotherapy finally relapse or progress after a period of time [45].

### 2.2. Goal: Tumor Cell Death

After the recognition of tumor neoantigens presented on the surface of cancer cells, activated T-cells are able to induce target cell apoptosis by the localized exocytosis of perforin, a homologous peptide to the complement component C9 which polymerizes in the target cell plasma membrane and form transmembrane channels [31]. T-cells also release serine proteases such as granzyme B which induces cell death in both caspase-dependent and -independent manner with the participation of BCL-2 proteins [31]. After Tumor Necrosis Factor (TNF)-ligand binding to the corresponding TNF-receptor expressed on tumor cells, activated T-cells can also indirectly trigger caspase activation and cell death [31]. 

Thus, although the principal reason why tumors would not respond to PD-1/PD-L1 blockade therapy is lack of recognition by T-cells due to the absence of tumor neoantigen expression and presentation [4], other different alterations such as apoptosis suppression or enhanced DNA repair are tightly related to treatment resistance [46]. In this regard, recent research on the molecular basis of cancer has demonstrated the role of altered protein kinases in tumor resistance and their powerful antiapoptotic activity against most therapeutic treatments including immunotherapies [31,43]. 

### 2.3. Receptor Kinases

The activation of a surface kinase receptor by its ligand or other stimulus leads to autophosphorylation in the kinase domain and represents the key initial step of signal transduction within the cell.

Receptor kinases are classified into two main families: The Receptor Tyrosine Kinase (RTK) family, constituted by cell-surface transmembrane proteins characterized by the presence of one extracellular ligand binding domain, a single transmembrane helix and a cytoplasmic region containing the tyrosine kinase activity and C-terminal regulatory regions, and the Receptor Serine/Threonine Kinase (RSTK) family, which are transmembrane proteins with extracellular ligand-binding domains and cytoplasmic kinase domains. Both RTK and RSTK families are further classified in different subfamilies based on kinase domain sequences (Table 2).

The presence of altered receptor kinases is a common phenotype in tumor cells (Table 2). Indeed, alterations in their activity, abundance, cellular distribution and/or regulation affect the functioning of signal transduction routes and can lead to the constitutive activation of down-stream kinases like PI3K/AKT, MAPK, EGFR or JAK/STAT [41] and different transcription factors that command cell survival [48] and trigger PD-L1 expression [4]. Accordingly, different studies have already reported the efficacy of receptor kinase inhibitors to enhance the response to PD-1/PD-L1 blocking therapies [32,33,34,35,36,37]. In this regard, the exhaustive preliminary study and the implementation of active monitoring [95] of patient kinase profile during the application of anti-PD-1/PD-L1 treatments may be valuable tools to identify those patients presenting altered receptor kinases and to select the most appropriate kinase inhibitor.

### 2.4. Non-Receptor Kinases

Extracellular signals are transduced within the cell as a result of the coordinated functioning of an intricate and complex network of cytoplasmic kinases controlling key cellular aspects. Because of this, alterations affecting receptor kinases function have a direct impact on cellular kinase transduction pathways.

On the other hand, under the pressure of the immune system and oncologic treatments, the activation of cellular mechanisms leading to apoptosis inhibition and cell survival represents a selective advantage for tumor cells over other cells [41]. Over time, this phenomenon, along with the typical genomic instability of tumor cells, increases the probability that these cells acquire new mutations affecting additional kinases down-stream receptor kinases. Thus, although the mutational burden of tumor cells may increase their antigenicity, it may also enhance their ability to evade treatment-induced immune responses [16] by interfering with the expected proapoptotic effect of PD-1/PD-L1 blocking therapies and receptor kinases inhibitors.

In this regard, along with receptor kinases, non-receptor kinases play a significant role in three aspects: by inhibiting apoptosis, by enhancing PD-L1 expression and by interfering with MHC expression.

#### 2.4.1. Non-Receptor Kinases and Apoptosis Inhibition

Due to its contribution to carcinogenesis, tumor progression and treatment resistance, intrinsic or acquired cell resistance to undergo apoptosis in response to stimuli constitutes one of the hallmarks of cancer [96]. Thus, and in consonance with their role as critical conveyers of extracellular signals, aberrant expression or dysregulation of cytoplasmic kinases leading to the constitutive activation of central survival pathways have been reported in cancer pathogenesis including oncogenesis, transformation, tumor cell survival and proliferation [97] (Table 3).

Apart from alterations affecting cellular central survival pathways commanded by PI3K/AKT/mTOR (phosphatidylinositol-3-kinase/protein kinase B/mTOR) pathway, MAPK (mitogen-activated protein kinase) pathway and PTEN (phosphatase and tensin homologue deleted on chromosome 10) phosphatase many other oncogenic and pro-survival kinases including c-Kit, c-MET, c-RET, s-SRC, S6 or AURK (aurora kinases) [97] are frequently mutated or altered in human cancer and are under intense study as targets for cancer treatment [43].

Provided that both the immune system and chemotherapy kill tumor cells by inducing apoptosis [38] and given the high prevalence and impact that alterations in cytoplasmic kinases have in human cancer in general and in apoptosis resistance in particular, alterations affecting these kinases may also cause the lack or partial response to PD-1/PD-L1 blockade and would also support previous kinase profiling studies and patient’s active monitoring [95] as valuable tools to enhance patient response to such treatments.

#### 2.4.2. Non-Receptor Kinases and PD-L1 Overexpression

PD-L1 over-expression also constitutes a major obstacle for anti-PD-L1 therapies, since apart from its strong immunosuppressive function over tumor-specific T-cells, PD-L1 also delivers intrinsic signals that enhance cell survival, regulate stress responses and confer resistance toward pro-apoptotic stimuli like interferons (IFNs) [49].

Although constitutive activation of RAS/RAF/MEK/ERK, mTOR and p38 pathways can trigger PD-L1 overexpression in cancer cells, PD-L1 expression is usually induced by different pro-inflammatory stimuli by the binding of transcription factors to its promoter, which explains why this protein is especially associated with inflamed tissues, including high infiltrated tumors [49]. In this regard, pro-inflammatory cytokines such as interferon gamma (IFNγ) produced by T-cells activates the intracellular non-receptor Janus tyrosine kinases (JAKs) leading to signal transducer and activator of transcription proteins (STATs) activation [49], translocation to the cell nucleus and induction of targeted genes transcription [113]. Accordingly, constitutive activation of STAT3, which directs the expression of anti-apoptotic and pro-survival genes, has been related to different malignancies including head and neck, breast and multiple myeloma [113]. JAK/STAT signaling is also dysregulated in hematological malignancies as well as in a large number of solid tumors contributing to oncogenesis, which has turned this pathway into a promising target for the development of new therapies [113] and a factor that should be considered when selecting candidate patients for immunotherapy.

Recent studies have shown that PD-L1 expression also depends on cell type and location and pathological situation [49] involving many other transcription factors such as SOX2 in hepatocellular carcinomas, STAT2 in human gliomas or STAT1 in multiple myelomas [49]. Since phosphorylation is one of the main post-translational modifications involved in the regulation of key cellular processes in general and signal transduction in particular [41], the role of protein kinases in such transcription factors is a field that deserves further study.

Nuclear factor-ĸB (NF-ĸB), the mediator of the TNFα signaling, significantly affects PD-L1 expression [49] as well. This transcription factor is involved in the regulation of the expression of genes involved in cell survival, proliferation, angiogenesis, metabolism, inflammation and cell adhesion/migration and, when constitutively activated, promotes tumorigenesis and drug resistance [114]. The inducible forms of NF-ĸB, which exist as inactive dimers in the cytosol of unstimulated cells through interactions with inhibitor of NF-ĸB proteins (IĸBs), is activated after IĸB phosphorylation by the IĸB kinase (IKK) complex provoking IĸBs ubiquitination and subsequent proteasomal degradation [115].

In this regard, recent studies suggest the potential utility of IKK inhibitors to enhance the response to cancer immunotherapies since NF-ĸB signaling can promote PD-L1 expression at both the transcriptional and post-transcriptional level [114]. However, despite the promising expectative of IKK inhibition, systemic delivery of IKK inhibitors is usually associated with high toxicities which implies a significative limitation and justifies additional research [114]. On the other hand, additional studies in this field have identified the Burton tyrosine kinase (BTK) as a target upstream NF-ĸB, which could become a promising therapeutic approach since different BTK inhibitors have already proven their safety and have been approved for the treatment of different hematological malignancies [114].

Although in NF-ĸB constitutive activation in solid tumors is usually due to chronic pro-inflammatory signaling within the tumor microenvironment and not to direct oncogenic mutations of its pathway components [114], preclinical studies have shown a role of *KRAS* (Kirsten Rat Sarcoma oncogene encoding for RAS) in NF-ĸB activation in which cell-autonomous feed-forward activation of PI3K/AKT and MAP kinases signaling pathways would be involved [114]. *KRAS* involvement in pathways related to both apoptosis inhibition and PD-L1 overexpression turn this oncogene into a potential prognosis marker that should be considered when stratifying patients for immunotherapy, as it has been already reported in patients with lung adenocarcinomas with *KRAS* mutations [116] and therefore, the rational use of kinase inhibitors targeting PI3K/AKT and MAP kinases in candidate patients for immunotherapy with *KRAS* mutant tumors should also be considered.

#### 2.4.3. Non-Receptor Kinases and MHC Expression

MHC class I (MHC-I) deficiency is an important mechanism of tumor escape from T-cell mediated immune responses found in approximately 40%–90% of human tumors derived from MHC-I positive tissues [117]. Indeed, provided that peptide presentation by MHC class I to be recognized by the T-cell receptor of CD8^+^T leads to the initiation of an adaptive immune response [118] decisive in the recognition and elimination of cancer cells, alterations affecting MHC-I transcription and expression will have an important impact on anti-PD-1/PD-L1 therapies and justify the need to find strategies that permit the recovery of MHC-I expression.

Transcription of *MHC-I* gene is tightly regulated at the transcriptional level by epigenetic mechanisms and different transcription factors that interact with conserved *cis*-acting regulatory promoter elements such as the enhancer A (bound by NF-ĸB, mediator of the TNFα pathway), IFN-stimulated response element (ISRE, mediator of IFNγ and JAK/STAT pathway) (bound by interferon regulatory factor IFR-family members), the SXY-module, the upstream-stimulatory factor (USF)-1, -2 and for the transcription factor Sp-1 [119]. For these reasons, alterations in kinases controlling these pathways can lead to a decreased expression in MHC-I and hence, to the resistant phenotype to PD-1/PD-L1 blockade. Thus, and for the reasons cited above, the combination of IKK and JAK/STAT targeting drugs in MHC-I deficient patients seems like a rational strategy to enhance the response to such therapies.

**Figure 5:**
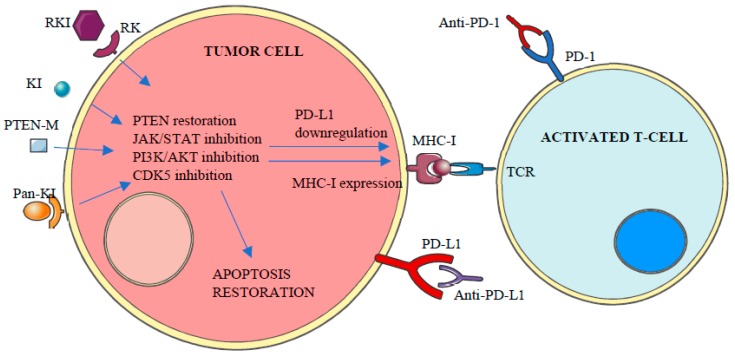
Protein kinase inhibitors can selectively inhibit the activity of their targeted altered kinase leading to PD-L1 downregulation and restoration of both apoptosis and MHC-I expression. Combination therapies with PD-1/PD-L1 blocking therapies will result in enhanced T-cell response against tumor cells. RK (receptor kinase), RKI (receptor Kinase Inhibitor), KI (kinase Inhibitor), Pan-KI (pan-kinase inhibitor), TCR (T-cell receptor). PTEN-M: PTEN modulator.

Similarly, several other kinases, including MAP2K1 (MEK1) and EGFR [120], have been identified to negatively regulate MHC and even cause checkpoint blockade upregulation [120], which endorses the use of kinase inhibitors in combination with immunotherapy (Figure 5).

### 2.5. Kinase Inhibitors and Anti-PD-1/PD-L1 Check-Point Inhibitors as Combination Therapy

The potential effectiveness of kinase inhibitors and anti-PD-1/PD-L1 therapy combos have already been explored in different studies. In this respect, a recent retrospective study has shown that the sequential therapy of anti-VEGFR2 tyrosine kinase inhibitor ramucirumab (Cyramza) with check-point inhibitors (nivolumab, pembrolizumab, atezolizumab) improved median overall survival of advanced NSCLC patients [121].

This approach has also been investigated in different clinical trials (Table 4), resulting in the recent Food and Drug Administration (FDA) approval of PD-1 inhibitor pembrolizumab in combination with the anti-VEGFR kinase inhibitor axitinib for the frontline treatment of patients with advanced renal cell carcinoma [32].

However, despite the more favorable side effect profile of kinase inhibitors when compared to conventional cytotoxic chemotherapy [43], and although the safety profile of PD-1/PD-L1 check-point inhibitors is consistent with that expected for immunologically mediated anticancer therapies, combination therapies can cause side effects such as pneumonitis, the affection of the skin, hormone glands, intestine, liver or kidney, including hepatitis, nephritis and kidney failure, among others [129]. For these reasons, these dose-limiting toxicities remain as one of the major limitations of such treatments and justify the need to continue investigating in this field.

## 3. Conclusions

Targeting the PD-1/PD-L1 axis has become a major pillar of immunotherapy and a great milestone which has helped increase the average life expectancy for cancer patients. However, the high rates of resistance acquisition, the large percentage of partial responders and the lack of durable responses remain as the great obstacles to the success of these therapies.

In the search for new weapons in the battle against cancer, numerous studies have revealed the role of altered kinases in different processes that can directly affect immunotherapies efficiency and the combined use with protein kinase inhibitors, along with protein kinase profiling studies during the design of biomarker panels, as reasonable approaches to enhance patient response. However, and despite the immediate clinical implications of these findings, there is still the need to evaluate the safety and efficacy of such combinations through the design of different clinical trials in which the most appropriate temporal administration protocol is also assessed.

Besides, given the complexity of kinase interrelations and dynamics, the use of kinase inhibitors targeting one single kinase usually results in the acquisition of resistance to such treatments [43] or even in the overexpression of PD-L1 as a result of the activation of alternate networks leading to STAT3 and ERK1/2 stimulation [130], a phenomenon that has already been reported in resistant non-small cell lung cancer [130] and that makes necessary a thorough preliminary proteomic study of patients in order to choose the more appropriate inhibitor combinations. In this regard, since kinase inhibitors would be especially useful when targeting kinases affected by chromosomal amplifications instead of mutations, new-generation sequencing and molecular profiling may be of prognostic and predictive value. Besides, since genomic instability of cancer cells can increase the risk of mutations affecting the conserved structure of kinase catalytic core and the subsequent loss of efficacy of kinase inhibitors, active monitoring of patients will also be required.

Still, and despite the encouraging prospects of kinase inhibition, it is necessary to be cautious and additional studies are still needed, since the use of kinase inhibitors may also affect PD-1 expression and lead to lymphocyte extenuation and death. On the other hand, although the inducers of cytokine release syndrome, or hypercytokinemia, remain unknown, it is a risk that should still be taken into consideration when applying these therapies.

## Figures and Tables

**Table 1:** Biomarker models for predicting efficacy of PD-1/PD-L1 inhibitors [10].

	Biomarker	Overview
Tumor Immune Microenvironment	PD-L1 expression measured by the proportion of positive/negative PD-L1 expressing tumor cell and/or immune cell.	Although PD-L1 expression is the most widely adopted predictor of patient response, conclusions from multiple trials are not consistent.
	Presence of absence of TIL.	Tumor immune microenvironment is classified into three subtypes (immune inflamed, excluded infiltrate, immune ignorance) [10]. Proposed as a valid biomarker in combination with PD-L1 expression status (PD-L1^+^TIL^+^/PD-L1^-^TIL^-^/PD-L1^+^TIL^-^/PD-L1^-^TIL^+^), transforming growth factor (TGF)-β or TIL derived interferon (INF)-γ.
Tumor Cell Intrinsic Features	Tumor mutational burden as a measure of accumulated mutations carried by tumor cells.	Proposed as a biomarker independent of PD-L1 expression that correlates with the increased production of neoantigens and elevated immunogenicity.
	Mismatch repair deficiency and microsatellite instability.	Related to the accumulation of mutations, elevated production of neoantigens, increased density of TIL, elevated tumor mutational burden, PD-L1 upregulation and enhanced immune response.
	Oncogenic driver mutations and other mutations.	Driver mutations affecting PD-L1 expression and subsequent TIL activation: epidermal growth factor receptor (EGFR), Kirsten rat sarcoma viral oncogene (*KRAS*), *ALK*.
Gut Microbiota	Cross-talk between gut microbiota and host immune system.	The modulatory effects of different bacterial species on the host immune system and anti-PD-1/PD-L1 therapies are under study.
Biomarkers in peripheral blood	Peripheral immune cells related to the inflammatory response (white blood cell count, neutrophil count, platelet count, lactate dehydrogenase, C-reactive protein, eosinophils, neutrophil-lymphocyte ratio, platelet-lymphocyte ratio).	Under study [11].
	Soluble PD-L1	Increased levels of soluble PD-L1 before treatment, which are usually caused by high tumor mutational burden, elevated alternative splicing and exhausted immune response, are associated with poor prognosis [10]. A rapidly increased soluble PD-L1 level after treatment is related to potent tumor-specific immune response and high partial response rate.
	Peripheral cytokine and other parameters reflecting the status of tumor immune microenvironment.	The value of different markers including prolactin, interleukin, interferon, tumor-derived vascular endothelial growth factor (VEGF), etc. is under study.
	Circulating tumor DNA and high PD-L1 circulating tumor cells	Patients with a high abundance of tumor PD-L1^+^ baseline tend to be more sensitive to therapy. Rapid eradication or decrease of circulating tumor DNA after treatment also correlates with robust anti-tumor effect.
Patient history	Patient previous history, pathological features and other predictors	Under study.
TIL: Tumor Infiltrating Lymphocytes		

**Table 2:** Receptor kinases classification and role in cancer.

	Receptor Kinase	Significance in Cancer
RTK	**I.** EGFR (EGF/HER/Erb) regulates epithelial tissue development and homeostasis [47] as well as cell proliferation, differentiation, survival, metabolism and migration [48].	*EGFR* mutation is a driver of tumorigenesis and biomarker of resistance to oncologic treatments [47]. Usually overexpressed in cancer cells, related to PD-L1 overexpression [49] and lack of response to anti-PD-1/PD-L1 immunotherapy [50,51].
	**II.** IGFR (InsR, IGF1R, IRR) key regulators of growth and energy metabolism [52], autophagy and apoptosis inhibition, DNA synthesis and amino acid uptake [53]. IGFR activation mediates the activation of PI3K signaling and NFAT and STAT6 transcription factors [54].	Participates in cancer development and progression [52] as well as in tumor chemoresistance associated with enhanced cell proliferation, apoptosis inhibition, regulation of ATP-binding cassette transporter proteins and extracellular matrix interactions [55].
	**III.** PDGFR (CSF-1R, KIT, FLT3) involved in angiogenesis [56] and regulation of cell growth and survival [57] by means of JAK/STAT, SRC and ERK activation [58].	Closely associated with tumor development, cell proliferation, metastasis, chemotherapy resistance, angiogenesis [56], immunosuppressive tumor microenvironment and apoptosis inhibition [41].
	**IV.** VEGFR (VEGFR-1, VEGFR-2, VEGFR-3) are key regulators of metabolic homeostasis, cell proliferation, migration, tubulogenesis, angiogenesis and lymphangiogenesis [41] through PKC-MAPK [59] and JAK/STAT signaling [60].	Central role in promoting endothelial cells survival, tumor vessels growth [61] and tumor-mediated immunosuppression [62].
	**V.** FGFR (FGFR1, FGFR2, FGFR3, FGFR4) is involved in angiogenesis and tissue development as well as in cell differentiation, survival and migration [63] by means of JAK/SRC-STAT activation [64].	Involved in carcinogenesis [65] through cell signaling deregulation, angiogenesis and resistance to cancer therapies [66].
	**VI**. PTK7/CCK4: This catalytically inactive receptor tyrosine kinase with a key role in Wnt pathway regulation and VEGF signaling [41] is essential for vertebrate cell motility during tissue morphogenesis [67]. PTK7 is involved in NF-ĸB activation by means of the PI3K/AKT signaling pathway [68].	Involved in tumorigenesis [69] and metastasis [67].
	**VII.** TRK/NTRK (TRKA, TRKB, TRKC) mediate proliferative and migration processes in neural systems [48].	*NTRK* gene fusions are oncogenic drivers of different types of cancers through the activation of different downstream pathways involved in the regulation of the transcription of genes involved in cell differentiation and survival [70]. Twenty-three percent of cancer patients presenting *NTRK* fusions express PD-L1 [71].
	**VIII.** ROR (ROR1, ROR2) act as alternative receptors and coreceptors of Wnt signals [41] with a role in cell proliferation, polarity and tissue maintenance [41] via PI3K/AKT/mTOR signaling pathway [72].	As non-canonical Wnt signaling mediator, this protein kinase has a dual role as a tumor suppressor or activator depending on tumor type or stage [41].
	**IX.** MuSK: The main organizer of subsynaptic specializations at the neuromuscular junction [73].	Activates down-stream signaling involved in cell proliferation, apoptosis, differentiation and tumorigenesis [73]
	**X.** HGFR (MET, RON): Regulate cell growth, cell motility and morphogenesis [74] and promotes PD-L1 expression in renal cancer cells [75].	Controls genetic programs leading to cell growth, invasion and apoptosis evasion [76].
	**XI.** TAM (AXL, TYRO3, MERTK): Required for the optimal phagocytosis of apoptotic cells in the mature immune, nervous and reproductive systems [77].	Involved in cell proliferation, survival, adhesion and migration [41]. PD-L1 expression correlates with both AXL and PI3K signaling in head and neck cancer [78].
	**XII.** TIE (TIE1, TIE2): Modulators of angiogenic and lymphangiogenic responses [41]. TIE2 is required for PI3K and AKT activation [79].	Activates down-stream signal transduction pathways related to cell survival and apoptosis inhibition [80].
	**XIII.** Eph (EphA1, EphA2, EphA3, EphA4, EphA5, EphA6, EphA7, EphA8, EphA10, EphB1, EphB2, EphB3, EphB4, EphB6): Modulators of cell proliferation, cell-cell attraction and repulsion, motility and sorting [81] by means of downstream regulation of SRC family kinases and PI3K [82].	These kinases are dependence receptors, playing dual functions as both oncogenes and tumor suppressors depending on the presence of their Ephrin ligands [81].
	**XIV.** RET: Involved in cell proliferation, neuronal navigation, cell migration and cell differentiation [41] through JNK/MAPK kinases activation [83].	Frequently mutated in different malignancies causing constitutive activation leading to cell survival and apoptosis inhibition [84].
	**XV.** RYK contains functional extracellular Wnt-binding domains and is implicated in Wnt signaling [41].	Although RYK is overexpressed in some types of cancer and correlated to worse survival, its exact function is unknown [85].
	**XVI.** DDR (DDR1, DDR2) modulates cell adhesion, proliferation and metalloprotease expression [41] through MAPK/ERK signaling pathway activation [86].	Regulate epithelial to mesenchymal transition, cell migration, invasion and survival, apoptosis inhibition and chemoresistance [87].
	**XVII.** ROS: Activates different signaling pathways involved in cell growth and survival [88] by means of PI3K/AKT pathway activation [88].	Aberrant expression of ROS (fusion isoform) is an important driver of different types of cancers [88].
	**XVIII.** LMR/LMTK (1, 2, 3): The exact function of these receptors is unknown [41].	LMTK3 is related to colorectal cancer progression [89] and plays a central role in breast cancer growth, metastasis and endocrine resistance [90].
	**XIX.** LTK (LTK, ALK): Endogenous ligands and precise roles of these receptors are unknown [41].	Genomic fusions affecting *ALK* gene is common in cancer and cause ALK constitutive activation leading to uncontrolled cell proliferation [41].
	**XX.** STYK (STYK1): Involved in cell proliferation, differentiation and survival [41] through MAPK, PI3K and AKT activation [91].	STYK aberrant expression is found in different malignancies including colorectal cancer [92].
RSTK	**I.** ALKs/ACVR (ALK1/ACVRLK1, ALK2/ACVRLK2, ALK3/BMPR1A, ALK4/ACVR1B, ALK5/TGFBR1, ALK6/BMPR1B, ALK7/ACVR1C): Cell-surface receptors for the TGF-beta superfamily of ligands. Survival of ALK-positive tumor cells is mediated by RAS/RAF/MEK/ERK pathway activation [93].	Defects in activin and TGF-beta signaling pathways are associated with the initiation and progression of the cancer phenotype [94].
	**II.** ActR2/ACVR2, ActR2B/ACVR2B, MISR2/AMHR2, BMPR2, TGFBR2: Form complexes and phosphorylate the kinase domain of RSTK type I.	
	**III.** TGFBR3: Accessory proteins that regulate the signaling of RSTK type I and II complexes.	

RTK: receptor tyrosine kinases: EGFR: epidermal growth factor receptor; IGFR: insulin growth factor receptor; InsR: insulin receptor; PDGFR: platelet-derived growth factor receptor; CSF-1R: colony stimulating Factor 1-Receptor; KIT: KIT proto-oncogene receptor tyrosine kinase; FTL3: FMS related tyrosine kinase 3; VEGFR: vascular endothelial growth factor receptor; FGFR: fibroblast growth factor receptor; PTK7: protein tyrosine kinase-like 7; CCK: colon carcinoma kinase; NTRK: neurotrophin receptor kinase; TRK: tropomyosin receptor kinase; ROR: receptor tyrosine kinase-like orphan receptors; MuSK: muscle-specific kinase; HGFR: hepatocyte growth factor receptor; MET: mesenchymal-epithelial transition factor; RON: receptour d’origine nantais; TAM: TYRO3-, AXL-, MER-TK receptors; TIE: tyrosine kinase with immunoglobulin-like and EGF-like domains or angiopoietin receptor; EphR: ephrin receptor; RET: rearranged during transfection; RYK: related to tyrosine kinase; DDR: discoidin domain receptor; TIE: tyrosine kinase receptor in endothelial cells; RYK: receptor related to tyrosine kinases; DDR: discoidin domain receptor; ROS: reactive oxygen species receptors; LMR: lemur receptor kinases; LTK: leukocyte tyrosine kinase; ALK: anaplastic lymphoma kinase; STYK: serine/threonine/tyrosine kinase 1. RSTK: serine/threonine kinases: ALKs: activin receptor-like kinases; ACVRL: activin A receptor type 1; BMPR: bone morphogenetic protein receptor; TGFR: transforming growth factor receptor; ActR2: activin A receptor type 2; AMHR: anti-mullerian hormone receptor; BMPR2: bone morphogenetic protein receptor type 2. JNK: c-JUN N-terminal kinase. MAPK: mitogen-activated protein kinase. mTOR: mammalian target of rapamycin. PKC: protein kinase C. ERK: extracellular signal-regulated kinase. NFAT: nuclear factor of activated T-cells. PI3K: phosphoinositide 3-kinase. NF-ĸB: nuclear factor kappa-light-chain-enhancer of activated B-cells. AKT: protein kinase B.

**Table 3:** Main non-receptor kinases related to apoptosis inhibition in human cancer.

	Kinase	Overview	Significance in Cancer
PI3K/AKT/mTOR pathway	PI3K	Transmits extracellular signals from receptor tyrosine kinases within the cell by catalyzing the production of PIP3, a phospholipid which triggers the activation of downstream signaling components such as AKT [97].	Directly implicated in the promotion of cell growth and survival [97]. Aberrant PI3K is implicated in 30%–50% of human cancers [97], PD-L1 overexpression and resistance to immunotherapy [4].
	AKT (AKT1, AKT2, AKT3)	One of the PI3K mediators, AKT phosphorylates and regulates the function of cellular proteins involved in metabolism, survival/apoptosis, differentiation and proliferation [98].	The most commonly dysregulated or mutated pathway in human cancer [99] contributes to PD-L1 overexpression [100] and apoptosis inhibition [101,102].
	mTOR (mTOR1, mTOR2)	Responsible for the phosphorylation and activation of AKT, mTOR is involved in the regulation of at least 800 different proteins [43].	Considered a master regulator of mammalian cell survival, proliferation and metabolism [103], aberrant expression or functioning of both mTOR1 and mTOR2 is found in up to 80% of human cancers [103,104] affecting tumor microenvironment and effector function [105].
MAPKs Pathway	RAS/RAF/MEK/ERK	Active ERKs phosphorylate different cytoplasmic and nuclear targets such as kinases, phosphatases, transcription factors and cytoskeletal proteins [106].	ERK pathway is deregulated in approximately one-third of all human cancers [106], with inhibitory effects on T-cell recruitment and function [4]. Activating mutations in RAS-RAF are frequent and key points to this pathway deregulation [106].
	JNKs	As master kinases, JNKs phosphorylate different transcription factors [106] and regulate physiological processes including inflammatory responses, morphogenesis, cell proliferation, differentiation, survival and death. In response to cellular stress, JNK binds to and phosphorylates tumor-suppressor p53 [106].	Persistent activation of JNK is involved in cancer development and progression [107].
	MAPK14 (p38)	p38 is activated by environmental stresses and inflammatory cytokines and phosphorylates different transcription factors [106]. p38 is required for TNFα and interleukin-1 expression during inflammatory responses [106].	A decrease in p38 activity plays an important role in cancer since it has a tumor-suppressive effect and plays a key role in the regulation of apoptosis, cell cycle progression, growth and differentiation [106]. Different chemotherapeutic agents require p38 activity for the induction of apoptosis [106]. Accordingly, the aberrant expression of MAPK has inhibitory effects on T-cell recruitment and function [4].
PTEN	PTEN	This dual-specificity protein and lipid phosphatase blocks PI3K signaling by inhibiting PI3P-dependent processes such as AKT membrane recruitment and activation [108], which results in the inhibition of cell proliferation and survival [43].	PTEN is one of the most frequently disrupted tumor suppressors in human cancer [43] whose loss has been related to resistance to anti-PD-1 blockade therapy [109].
JAK/STAT	JAK/STAT	The JAK/STAT signaling pathway is the principal signaling mechanism for different cytokines and growth factors, being involved in processes such as immunity, cell proliferation, differentiation, migration and apoptosis [25].	Mutations in *JAK1, JAK2* genes are associated with primary and acquired resistance to PD-1 blockade therapy [110].
	STK11/LKB1	This tumor suppressor serine/threonine kinase controls the activity of AMPK family members, playing a key role in cell metabolism, cell polarity, apoptosis or DNA damage response [111]	In advanced non-squamous lung cancer, somatic STK11/LKB1 mutations confer resistance to PD-L1 checkpoint inhibitors as monotherapy or in combination [112].

PI3K: phosphatidylinositol-3-kinase; PI3P: phosphatidylinositol (3,4,5)-triphosphate; AKT: protein kinase B; mTOR: mammalian target of rapamycin; MAPKs: mitogen-activated protein kinases; RAF: rapidly accelerated fibrosarcoma; ERK: extracellular-signal regulated kinase; MEK: MAPK/ERK kinase; JNK: c-Jun N-terminal kinase; TNF: tumor necrosis factor; PTEN: phosphatase and tensin homologue deleted on chromosome 10. AMPK: AMP-activated protein kinase. STK11/LKB1: serine/threonine kinase 11/liver kinase B1.

**Table 4:** Approved PD-1/PD-L1 check-point inhibitors and combination studies with kinase inhibitors.

	Name	Overview	Combination
PD-1 Inhibitor	Pembrolizumab (Keytruda, Merck and Co., Inc.)	FDA approved in 2014 for the treatment of melanoma and subsequently approved for metastatic NSCLC and head and neck squamous cell carcinoma with positive PD-L1 expression and no EGFR or ALK alterations [122].	FDA approved in combination with the VEGFR kinase inhibitor axitinib (Inlyta) for the frontline treatment of patients with advanced RCC [32].
	Nivolumab (Opdivo, Bristol-Myers Squibb Co.)	FDA approved in 2014 for the treatment of melanoma and subsequently approved for SCLC, RCC and Hodgkin’s lymphoma [123]. Nivolumab efficacy on EGFR mutation-positive NSCL cancer patients is limited [124].	Phase I study show that nivolumab plus anti-EGFR tyrosine kinase inhibitor erlotinib is tolerable, with durable responses in NSCLC patients with altered EGFR [33]. Combination with antiangiogenic tyrosine kinase inhibitors (sunitinib, pazopanib) is also showing encouraging preliminary antitumor activity across different tumor types [34], although high-grade toxicities may limit this approach and make it necessary careful selection of the antiangiogenic component and dose [34].
	Cemiplimab (Libtayo, Regeneron Pharmaceuticals, Inc.)	FDA approved in 2018 for the treatment of CSCC or locally advanced CSCC who are not candidates for curative surgery or curative radiation [125].	Several case reports of EGFR inhibitors and single arm, prospective studies of cetuximab and gefitinib in patients with high-risk CSCC have reported objective responses [35].
PD-L1 inhibitor	Atezolizumab (Tecentriq, Roche Genentech)	FDA approved in 2016 for urothelial carcinoma and NSCLC in patients with strong PD-L1 expression and without EGFR or ALK alterations [126].	Benefits of atezolizumab in combination with anti-VEGF bevacizumab and pemetrexed and carboplatin chemotherapy drugs is under single arm phase 2 clinical trial [36].
	Avelumab (Bavencio, Merck Sernon-Pfizer)	FDA approved in 2017 for the treatment of metastatic merkel-cell carcinoma.	Results of a randomized, phase 3 study show avelumab in combination with the anti-VEGFR tyrosine kinase inhibitor axitinib (Inlyta) as a potential new first-line standard of care for patients with advanced RCC [37].
	Durvalumab (Imfinzi, AstraZeneca)	FDA approved in 2018 for the treatment of urothelial carcinoma and unresectable non-small cell lung cancer after chemoradiation [127].	Early clinical activity of durvalumab in combination with anti-EGFR tyrosine kinase gefitinib has been reported in a phase I study with NSCLC patients [127]. Efficacy of durvalumab in combination with vistusertib mTOR inhibitor, AZD4547 anti-FGFR tyrosine kinase inhibitor in patients with muscle invasive bladder cancer is under clinical trial [127] Recruitment of a phase III open-label trial investigating the efficacy of third-generation EGFR tyrosine kinase inhibitor osimertinib (Tagrisso) in combination with durvalumab in NSCLC patients was terminated early because of increased incidence of interstitial lung disease-like events [128].

CSCC: cutaneous squamous cell carcinoma, RCC: Renal Cell Carcinoma, NSCLC: non-small cell lung cancer.
